# Generation of Liquid Crystal Elastomer Fibers via a Wet Spinning Technology with Two-Stage Crosslinking

**DOI:** 10.3390/polym17040494

**Published:** 2025-02-13

**Authors:** Lukas Benecke, Sina Anna Schwingshackl, Peter Schyra, Chokri Cherif, Dilbar Aibibu

**Affiliations:** 1 Institute of Textile Machinery and High Performance Material Technology, TU Dresden, 01069 Dresden, Germanypeter.schyra@tu-dresden.de (P.S.); chokri.cherif@tu-dresden.de (C.C.); dilbar.aibibu@tu-dresden.de (D.A.); 2 Centre for Tactile Internet with Human-in-the-Loop (CeTI), TU Dresden, 01062 Dresden, Germany

**Keywords:** liquid crystal elastomer fiber, actuator, wet spinning

## Abstract

Liquid crystal elastomers (LCE) are a promising material to achieve reversible actuation while being able to perform work, showing great potential as artificial muscles in soft robotics and medical technology. Here, a wet spinning process to prepare liquid crystal elastomer fibers (LCEF) with reversible actuation capability is presented. Furthermore, we demonstrate the ability to process side-chain liquid crystal (LC) 4-Methoxyphenyl 4-(3-butenyloxy)benzoate (MBB) into a fiber, enlarging the material variance available in this field. The wet spinning process is presented and discussed in terms of spinning parameters and their influence on fiber properties, especially LC orientation. Moderate draw ratios of up to 2.3 enable highly oriented mesogens (f = 0.64), enabling the contractile behavior. The generated MBB-based LCEF show low activation temperature (54.52 °C), temperature-dependent mechanical properties, reversible contraction behavior while lifting up to 140 times their own weight and are able to perform work of up to 3.857 J kg^−1^. Actuation properties are compared with human skeletal muscle, and possible strategies of further enhancing the LCEF performance are discussed. The generated data show promising features of the LCEF for use as artificial muscle fibers in medical applications, e.g., prosthetics and artificial cardiac tissue.

## 1. Introduction

Liquid crystal elastomers (LCE) are a class of materials that combines the anisotropic order of liquid crystals (LC) with the elasticity of lightly crosslinked polymeric networks (elastomers) [1]. The LC used for this material are typically simplified as rod-like molecules and directly linked to the elastomeric backbone, either within the polymeric main chain or as a side chain via crosslinks [2,3,4]. Like in liquid crystal displays (LCDs), LC in LCE possess the ability to reorientate when exposed to external stimuli, e.g., electric/magnetic fields or heat [3,5,6]. This reorientation is transferred to the elastomeric backbone due to the rod-like nature of the LC. This results in a bending of the polymer chains and, provided that the rod-like LC were oriented, in a macroscopic contraction of the LCE [7]. After the external stimulus is omitted, the elastomeric backbone can restore the original shape due to preserved crosslinks [8]. This ability to contract reversibly has made LCE a promising material for soft actuators. Sophisticated material designs and setups enable LCE to achieve multidirectional actuation, oscillation, re-programmability, as well as complex actuation with high degrees of freedom, as shown by Saeed et al., amongst others [9,10,11,12].

Especially for medical applications, LCE offer great potential to mimic human muscles. To not only emulate its function but, furthermore, to be able to recreate building principles, the fibrous morphology of muscle needs to be considered. Therefore, a continuous spinning process to create liquid crystal elastomer fibers (LCEF) is of great importance. Furthermore, fiber spinning offers great potential to increase molecular orientation through shear forces inside the spinning nozzle and fiber drawing, enhancing contraction and mass-specific work. Since de Gennes proposed the theoretical potential of LCEs as artificial muscles in 1975, research interest in this material system awoke [13]. Küpfer and Finkelmann experimentally confirmed de Gennes’ theoretical work in 1991, fabricating a nematic LCE foil, i.e., elastomers with permanent, macroscopically uniform alignment by incorporation of LC in a poly(methylhydrosiloxane) (PMHS) backbone via crosslinking [14]. A one-pot method including a two-step crosslinking technique was developed that enabled shape fixation (first crosslinking) and fixation of nematic (anisotropic) alignment after stretching (second crosslinking). The first fiber-like structure was achieved in 2003 by Naciri et al., who fabricated LCEF by dipping a metallic tweezer into a specially synthesized polymer-LC melt combined with MDI crosslinker and “[…] pulling the mixture with it as quickly as possible” [15]. This hand-drawing procedure requires extensive polymer synthesis and results in small fiber segments of uncontrolled thickness, which is unsuitable for further practical application. In 2007, Krause et al. utilized electrospinning of a main chain LCE to generate oriented fibers [16]. However, these fibers were generated in a randomly oriented fiber mat. Nevertheless, even though a high nematic orientation was achieved, no contraction behavior was observed. Furthermore, the main chain LCE had to be synthesized prior to electrospinning since no commercially available main-chain LCE were known. In 2011, Ohm et al. presented a microfluidic approach in which droplets of LCE solution were forced through a capillary utilizing a co-flowing stream of silicon oil and polymerized via UV irradiation, yielding anisotropic LCE particles or fiber fragments according to capillary diameter [17]. LC orientation was achieved via shear forces of the liquid flow and capillary walls. According to flow conditions and capillary diameter, contraction or expansion of the LCE particles was observed upon stimulation above nematic–isotropic transition temperature (T_NI_). Even though this method yielded promising results, no continuous fiber preparation can be achieved.

Recently, new developments in LCEF spinning were published, showing advancements compared to these early experiments. Since 2022, several publications presented methods to generate LCEF using templates like PTFE tubes to achieve noncontinuously spinnable fibers [18,19,20,21,22]. Here, a demolding step is necessary after form fixation inside the template, making it a two-step process with no up-scaling capability. Wang et al. published an alternative fabrication method, where a pre-crosslinked LCE solution was extruded onto a PTFE roller, stretched onto a second roller and UV-cured [23]. Based on our comprehensive research, this is the first reported instance of continuous LCEF spinning. Nevertheless, this method has a significant drawback caused by the pre-crosslinking step of the LCE solution to obtain sufficiently high viscosities. Wang et al. reported that the spinning solution needed to react at room temperature for 20 min before extrusion was started and afterwards the extrudate was cured on a roller for another 2 h at room temperature [23]. These processing steps are susceptible to errors since slight deviations in temperature, time and LCE solution composition can strongly vary viscosity and, therefore, fiber properties, as known from our experimental observations. Wu et al. presented a similar spinning method, resulting in identical drawbacks [24]. Hou et al. increased viscosity by evaporating large amounts of the solvent prior to extrusion, thus generating a highly viscous LCE solution that was stable enough to experience shear orientation and could be drawn in a dry spinning setup prior to UV crosslinking [25]. Again, a processing step was susceptible to errors, with large variations in resulting viscosity and, therefore, fiber parameters.

As can be seen in the examples mentioned, previous approaches to produce LCEF either lack the reproducibility for continuous fiber production, require complex material synthesis, or lack the capability for up-scaling. Furthermore, all presented research works use identical mesogens (RM82 and/or RM257) for spinning LCEF [18,19,20,21,22,23,24,25]. Other LC materials have not yet been manufactured as contractile fibers. Li et al. presented a different material composition with promising actuator properties, utilizing the side-chain mesogen 4-Methoxyphenyl 4-(3-butenyloxy)benzoate (MBB) [26,27]. Their actuator foils displayed reversible contractions of ~28% while generating stresses of 14 kPa. Furthermore, this LCE has a moderately low nematic–isotropic transition temperature, rendering it suitable for applications close to the human body. Though, MBB-based LCEE could not be manufactured as fibers. Here, a wet spinning process is proposed, which enables continuous fiber production of MBB-based LCE with reversibly contractile properties and that possesses high up-scaling potential Actuator properties of the resulting LCEF are characterized with regard to potential application as artificial muscles.

## 2. Materials and Methods

### 2.1. Materials

Liquid crystal 4-Methoxyphenyl 4-(3-butenyloxy)benzoate (MBB) and crosslinker 1,4-Bis(10-undecen-1-yloxy)benzol (11UB) were synthesized by GenoSynth GmbH (Berlin, Germany). Polydimethylsiloxane (PDMS), Polymethylhydrosiloxane (PMHS), Dichloro(1,5-cyclooctadien)platinum(II) (catalyst), and toluene (≥99.5%) were purchased from Merck KGaA (Darmstadt, Germany). Silicone oil (PMHS, 350 cSt) was purchased from VWR International, LLC (Darmstadt, Germany). All materials were used as delivered without further purification.

### 2.2. Preparation of LCE-Spinning Solution

Catalyst solution was prepared by adding 25 mg of platinum catalyst into 20 mL of toluene and was stirred for at least 24 h to achieve homogenization. LCE spinning solution was prepared by mixing MBB (870 mg, 2.91 mmol), 11UB (105 mg, 0.255 mmol), and PDMS (381 µL, 0.015 mmol) in 1.5 mL of toluene under constant stirring, generating a molar LC/crosslinker ratio of approx. 1:0.09. After a clear, yellowish solution was formed, 0.6 mL of PMHS (2.7 mmol) and 1.845 mL of catalyst solution were added. The solution was then homogenized under ultrasonic treatment for 10 min and placed in a desiccator connected to a vacuum pump (VACUSAFE comfort, INTEGRA Biosciences GmbH, Biebertal, Germany) for degassing at 0.3 bar for 10 min.

### 2.3. Characterization of Crosslinking Kinetics

Isothermal rheological measurements of the spinning solutions were performed to characterize the crosslinking kinetics. At first, temperature was set to 25/60/70/80/90 °C using a temperature controller (HAAKE UTMC, Thermo Electron Karlsruhe GmbH, Karlsruhe, Germany). Approx. 1 mL of freshly prepared spinning solution was placed on an aluminum plate (P35 ALU) and put into the rheometer (HAAKE MARS, Thermo Electron Karlsruhe GmbH, Karlsruhe, Germany). An oscillating measuring mode (1 Hz) was used and viscosity was measured for 1 h. The resulting time-dependent viscosity was defined as a measure of the progression of crosslinking. Triplets for each temperature were used. Mean viscosity at 25 °C (room temperature) and after reaching a plateau were defined as 0% and 100% of crosslinking density, respectively. To better evaluate the generated data, a fit was applied using Equation (1), where η and t are viscosity and time, respectively.

(1) $$\mathrm{lg}\left(\eta \right)=\frac{a}{1+c•10^{-t•d}}+b$$

Parameters a, b, c, and d were defined for each data set and correspond to the difference between η_max_ and η_min_, η_min_, timely setoff of crosslinking initiation, and slope (crosslinking speed), respectively.

### 2.4. LCE-Fiber Spinning

The spinning solution was transferred directly after preparation into a syringe (Omnifix Solo, 10 mL, B. Braun SE, Melsungen, Germany) and was connected to a 21G needle (Sterican^®^ blunt, 0.8 × 22 mm, B. Braun SE, Melsungen, Germany) via a 100 mm perfusion line (Original Perfusor^®^ Line, 1.0 × 2.0 mm, PE, 10 cm, B. Braun SE, Melsungen, Germany). The syringe was mounted onto a syringe pump (IPS14, Inovenso Inc., Cambridge, MA, USA). The extrusion speed was set to 48 mL h^−1^. The needle was thoroughly placed into a silicone-oil-filled spinning bath at an angle of approx. 30°, beneath fluid level. The spinning bath temperature was set to 90 °C (2× Arex heating plate, VELP Scientifica, Usmate, Italy). The silicone oil was circulated via a peristaltic pump (DOSE IT, INTEGRA Biosciences GmbH, Biebertal, Germany), generating a current of 12 L h^−1^, assisting fiber haul-off. After being solidified, fibers were taken up by a coil (ø = 25 mm), located above the heated spinning bath to achieve passive heating for 2nd crosslinking, at a speed of 1.15 m min^−1^. The whole setup and relevant spinning parameters can be seen in Figure 2. Finally, the as-spun fibers were gradually drawn via three additional coils, where the last coil had a speed of 2.65 m min^−1^, generating an overall drawing ratio of 2.3.

### 2.5. Mesogen Orientation

For characterization of mesogen orientation in the LCEF, X-ray diffraction (XRD) scans were performed using an Empyrean (Malvern Panalytical Ltd., Worcestershire, UK). The scans were operated with a copper X-ray tube, 45 kV, 40 mA, and θ/θ-goniometer setup. For sample preparation, LCEF were cut into small pieces, led side by side, and fixated with mylar foil (Figure 4). At first, a 2θ scan was performed to determine a suitable angle for φ scans. These scans were then performed using the determined 2θ ± 0.5°, while the sample stage was rotated ± 180°. The Full Width Half Maximum (FWHM) of the resulting peak was used to calculate the orientation index f_c_ using Equation (2) [28]:

(2) $$f_{c}=\frac{180{}^{\circ}-\beta }{180{}^{\circ}}$$

where β is determined from the FWHD of the azimuthal direction of the reflection of interest.

### 2.6. Determination of Nematic–Isotropic Transition Temperature

The nematic–isotropic transition temperature (T_NI_) was characterized by differential scanning calorimetry (DSC) on a Q2000 (TA Instruments Inc., Eschborn, Germany). Samples of the drawn fiber were thermodynamically analyzed from 20 °C to 120 °C. The heating rate was set to 2 K min^−1^. T_NI_ was defined as peak maximum and middled using triplets.

### 2.7. Characterization of Mechanical Properties

Uniaxial tensile tests were performed on a Zwicki Junior (ZwickRoell GmbH & Co. KG, Ulm, Germany) to determine the elastic moduli, tensile strength, and elongation at break of the LCEF at room temperature and above T_NI_ at 60 °C (according to DSC measurements) for 10 specimen each. A 100 N load cell and barometric clamps (1.5 bar) were used to measure filaments of 100 mm in length. A preload force of 0.05 N was applied and the tests performed with a dislocation speed of 2 mm min^−1^. Elastic moduli were determined between strains of 0.05–0.25%. To determine the fiber diameter for moduli calculation, prior to the tensile tests, scanning electron microscopy images were generated and evaluated via image analysis.

### 2.8. Characterization of Contractile Capacity and Specific Work

To characterize contractile capacity and specific work, firstly, LCEF specimens of 120 mm in length were weighed and measured. Afterwards, increasing weights (starting at 3.5 g) were thoroughly attached onto the LCEF (Figure 7A), which were placed into a heating chamber and heated to 100 °C (well above T_NI_ offset according to DSC to assure full contraction). The custom-made fiber contacts assured no slipping of the LCEF during heating. After the temperature was reached, the length of the LCEF was measured. Specific work was calculated, using Equation (3), where m*_W_* is the mass of the attached weight, a is the gravitational acceleration (9.81 m s^−2^), Δs is the length difference of LCEF after contraction, and m*_LCEF_* is the mass of the LCEF.

(3) $$W=\frac{m_{W}•a•\Delta s}{m_{\mathrm{LCEF}}}$$

This procedure was repeated with increasing weight until LCEF breakage occurred. Regarding the calculation of specific work, the mass of the whole 120 mm LCEF was used without deduction of the parts solely used for fixation of the fiber or weights.

## 3. Results

### 3.1. Crosslinking Kinetics

**Figure 1:**
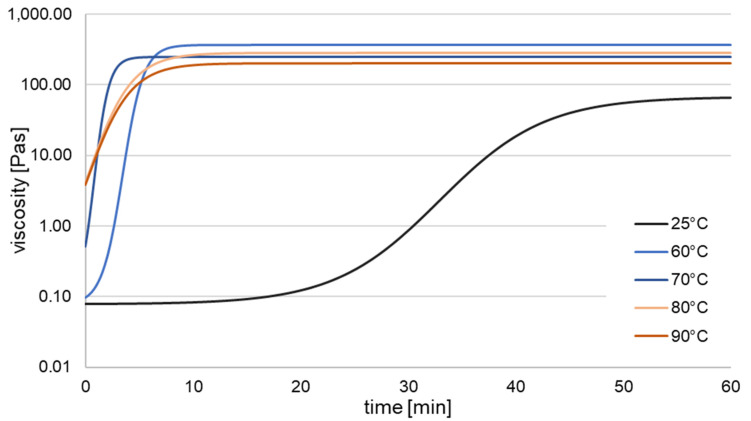
Viscosity (averaged fit) of the LCE spinning solution over time during crosslinking at different temperatures, *n* = 3.

At room temperature, freshly prepared spinning solution had a viscosity of 0.078 ± 0.007 Pa s (Figure 1). This value was correlated to a crosslinking density of 0%. Depending on the applied temperature, after some time, viscosity values rose, reaching a value of 292.111 ± 114.962 Pa s or 100% crosslinking density. For room temperature rheology measurements, no stable η_max_ was reached after 60 min. With increasing temperature, a decrease in η_max_ could be observed (from 382.566 ± 105.796 Pa s at 60 °C to 209.389 ± 47.654 Pa s at 90 °C), showing a temperature-dependent softening of the LCE, as also shown in the tensile tests. Furthermore, initiation time (set-on) of crosslinking was decreased for increasing temperature, as can be seen in Figure 1 and Table 1. For 70 °C and higher, crosslinking starts immediately when rheology experiments were conducted. Negative values result from a timely offset (approx. 5–10 s) between sample filling (onto preheated rheology plate) and data acquisition. Nevertheless, especially for higher temperatures, crosslinking already started at this time, enhancing the initial viscosity of these specimens. This behavior of immediate solidification after heat treatment was desired to achieve shape stability during the wet spinning process.

### 3.2. LCE-Fiber Spinning

**Figure 2:**
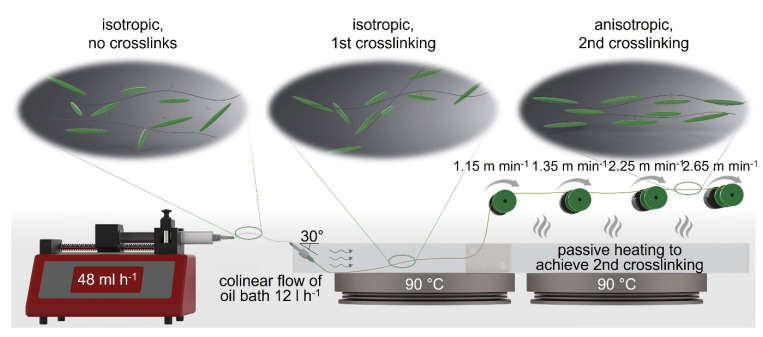
Spinning setup and mesogen orientation according to process progression.

To realize wet spinning of LCE, a lab-scale spinning setup was developed (Figure 2; detailed description in Materials and Methods Section 2.4). Rheological analysis indicated that crosslinking occurred immediately upon exposure of the spinning solution to temperatures above 70 °C. However, during fiber spinning, temperatures below the threshold value of 90 °C resulted in random ramification of the spinning solution prior to solidification. Differences between rheological measurements and empirical process observations may result from additional energy input caused by oscillation. Higher temperatures lead to crosslinking in the perfusor line and ultimately to clogging of the needle. The flow generated by the peristaltic pump was crucial for ensuring stable fiber formation, fiber take-off, and preventing needle clogging.

**Figure 3:**
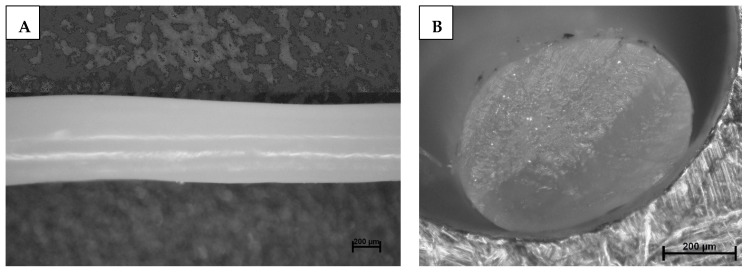
Image of as-spun LCEF surface (**A**) and elliptical cross-section (**B**).

The fibers exhibit relatively low mechanical strengths after the first thermal crosslinking due to low degrees of crosslinking and high degrees of swelling with toluene, both of which are necessary for fiber programming through subsequent drawing [14,29]. Therefore, fiber uptake has to proceed cautiously to avoid damaging the as-spun LCEF. To attain high drawing ratios for mesogen orientation, mechanical stress on the fibers was reduced through multiple drawing steps (up to three separate steps). A drawing ratio of 2.3 resulted in homogenous fibers with an elliptical cross-section (Figure 3) and a fineness of 360.38 ± 79.17 g km^−1^ (tex).

**Figure 4:**
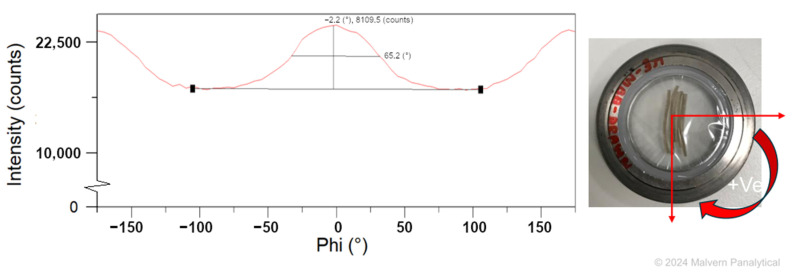
Phi scan of LCEF showing orientation of mesogens in fiber axis; inlay shows sample preparation with mylar foil.

To evaluate mesogen orientation, XRD scans were performed. A 2θ scan showed a distinct peak (separate from the utilized mylar foil) at 12.3°, which was used for the subsequent φ scan. The results of this φ scan are shown in Figure 4. A FWHM of 65.2° was determined for the LCEF, resulting in an orientation index of 0.64. Therefore, a clear orientation of the mesogens parallel to the fiber axis can be derived.

### 3.3. Nematic–Isotropic Transition (T_NI_) of LCEF

**Figure 5:**
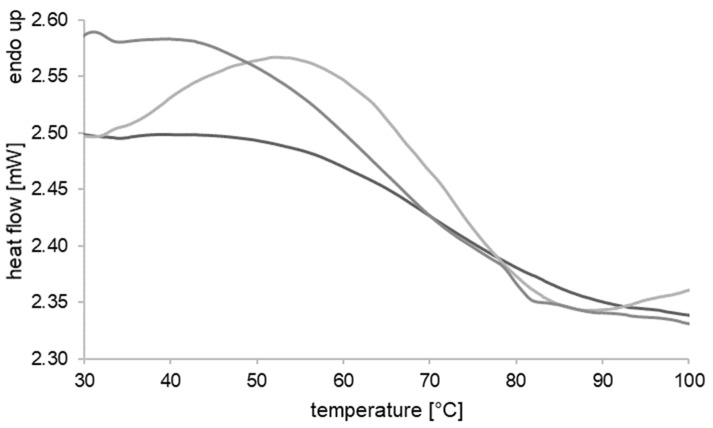
DSC measurements of LCEF with broad endothermic peaks characterizing the nematic–isotropic transition.

Differential scanning calorimetry (DSC) analysis showed broad endothermic peaks during the heating of drawn fiber fragments (Figure 5). The nematic–isotropic transition temperature T_NI_ was determined by evaluating the peak maximum. For the prepared LCEF, this was at 54.52 ± 2.26 °C. Moreover, experimental determination of the contractive capability demonstrated that LCEF possess the ability to continuously adjust their degree of contraction until maximum contraction is reached. Therefore, the onset (33.20 ± 0.47 °C) and offset (88.14 ± 4.03 °C) temperature of the endothermic peak hold significant relevance in characterizing the contractile behavior of LCEF.

### 3.4. Mechanical Properties

**Figure 6:**
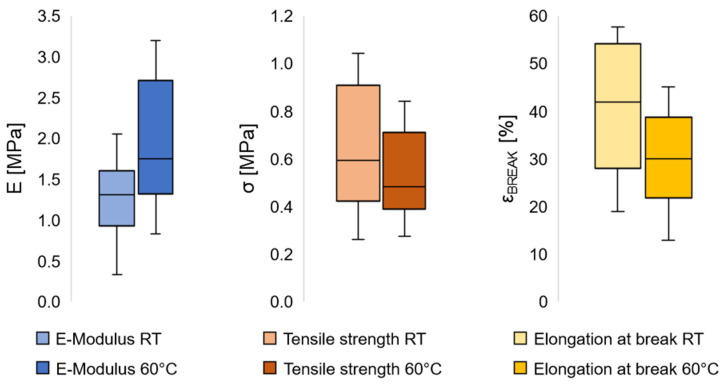
Elastic moduli, tensile strength, and elongation at break of LCEF tested at room temperature (RT) and above T_NI_ (60 °C), *n* = 10.

Mechanical properties of the LCEF were determined to evaluate their application as potential artificial muscles. Since the mechanism of contraction is based on thermally induced molecular reorientation of mesogens, differences in the mechanical properties are expected below and above T_NI_. At room temperature, LCEF showed an elastic modulus of 1.26 ± 0.46 MPa and a tensile strength of 0.64 ± 0.24 MPa or 0.058 ± 0.022 cN tex^−1^ (Figure 6). Above T_NI_, an increase in the E-modulus and decrease in tensile strength to 1.94 ± 0.75 MPa and 0.52 ± 0.17 MPa (0.047 ± 0.016 cN tex^−1^) was observed, respectively. Elongation at break decreased above T_NI_ from 40.6 ± 13.2% to 29.4 ± 9.7%. Temperatures above T_NI_ result in a loss of mesogen orientation as well as elongation of the elastomeric backbone [30]. Therefore, a decrease in the tensile strength was expected. On the other hand, the increase in E-modulus and decrease in elongation at break seemed counterintuitive. In LCE, contraction is entropy-driven [30]. When heated above T_NI_, the thermal energy enables the mesogens to rotate/reorientate and achieve a state of maximized entropy. When being drawn during a tensile test, the elastomeric backbone will be stretched and polymer chains aligned, decreasing the entropy. The effort of the LCE to maximize their entropy above T_NI_ thus forms a counterforce to the deformation, which might cause the increase in E-moduli and decrease in elongation at break in LCEF.

### 3.5. Contractile Capacity and Specific Work

**Figure 7:**
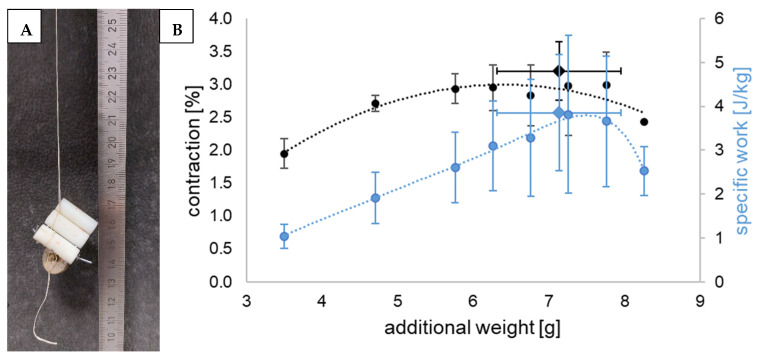
Picture of the measuring setup (**A**); contraction (black) and mass-specific work (blue) of LCEF moving additional weights while being activated at 100 °C in a heating chamber (**B**); circles represent mean values (*n* = 4); diamonds show maximum values; bars represent standard deviation.

To characterize the contractile capacity and performed mass-specific work of single LCEF, weights were attached to the fibers before activation in a heating chamber (Figure 7A). The analyzed LCEF were able to contract while moving up to 8.26 g (weighted plate + attachment), demonstrating their capability to lift 140 times their own weight (0.058 ± 0.013 g). Higher weights led to fiber breakage after heating as the fibers softened above T_NI_ (as seen in tensile tests). LCEF contraction was observed to varying degrees in all samples (Figure 7B; Table 2). Starting at 3.5 g (weight of the fiber contacts without weighted plates), ~2% contraction could be achieved. The contraction increased with increasing weights, reaching a plateau at approx. 3%. Therefore, to maximize contraction under load, the prestress of the LCEF must be carefully considered. Mass-specific work increased linearly with increasing weight to 3.857 ± 1.318 J kg^−1^ while lifting 7.14 ± 0.82 g and achieving contractions of 3.21 ± 0.44%, respectively. Afterwards, the performed work decreased due to less contraction, even though higher weights were moved.

## 4. Discussion

The LCEF showed an activation temperature (T_NI_) of ~55 °C. It was demonstrated that the material softened when heated above T_NI_, resulting in a reduction in tensile strength. In contrast, the E-modulus increased while elongation at break decreased above T_NI_, which could be attributed to LC’s effort to remain an isotropic conformation when stretched above T_NI_. The LCEF showed a moderate mesogen orientation after spinning as shown in XRD measurements. When undergoing the nematic–isotropic transition, the LCEF were able to lift up to 140 times their own weight while performing load-dependent contractions of up to 3%, underlining the importance of prestress for practical applications. Under load, they performed a mass-specific work of up to 3.9 J kg^−1^. Therefore, LCEF show promising features as artificial muscle fibers, potentially being used in medical applications as prosthetics or to achieve pumping functions similar to cardiac tissue.

The material composition of 4-Methoxyphenyl 4-(3-butenyloxy)benzoate (MBB, liquid crystal), 1,4-Bis(10-undecen-1-yloxy)benzol (11UB, crosslinker), and Polydimethylsiloxane (PDMS, elastomeric backbone) is the object of multiple studies in the literature, although only manufactured as films [27,31,32,33,34]. These LCE films achieve specific work ranging from 0.21 J kg^−1^ [34] to 11.45 J kg^−1^ [32], averaging at 3.46 J kg^−1^ [27,31,32,33,34] (values of specific work were not presented in these works; calculations were performed using LCE-film dimensions, contraction rate, additional weight, mass of LCE film, contraction stress, and/or, if not given, density of MBB–11UB–PMHS -blend of ~1100 kg m^−3^ as determined in this work). Therefore, LCEF prepared in this work are comparable to these LCE films in regard to specific work capacity. Studying these publications, it became apparent that all of these LCE films achieved contraction rates of about 33%, approximately seven times more than the prepared LCEF could achieve. Since contractile capability in LCE is closely linked to mesogen orientation [30], further studies will focus on increasing the orientation index (currently 0.64) through modifications of the spinning process. Further focus will be put into the mesogen concentration in the network, which was decreased compared to literature composition due to spinnability of the material. Elevating the contractile capacity of LCEF towards literature values will potentially enhance the performed work by a factor of up to 7 towards 27 J kg^−1^. According to Madden et al., mass-specific work of mammalian muscle ranges from 7.71 J kg^−1^ to 38.57 J kg^−1^ [35]. Therefore, further enhancing contractile capability of LCEF will enable these fibers to perform specific work in the upper range of human muscle. Regarding mechanical properties, advancements are needed to biomimetically replace human muscle. While the tensile strength of LCEF is considerably higher (0.64 ± 0.24 MPa vs. 0.1–0.35 MPa), the E-modulus of human muscle is at least tenfold higher (10–60 MPa) [35]. Again, increasing the order parameter of LCEF through modified spinning parameters and material composition is assumed to enhance the mechanical properties, enabling the application of LCEF as artificial muscles.

## 5. Conclusions

For the first time, a successful wet spinning process was developed that enables the reproducible generation of liquid crystal elastomer fibers of any known composition. Furthermore, LCE based on MBB have been processed into fiber form for the first time. Due to their capability to contract reversibly while lifting up to 140 times their own weight, these fibers are well suited for various applications in soft robotics, architecture (e.g., adaptive, self-sensing shading elements), and particularly in medical technology. Here, LCE could serve as support or replacement for muscles, for example, in prosthetics, orthotics, or in development of innovative cardiac support systems.

## Figures and Tables

**Table 1:** Characteristic values of solidification of the LCE spinning solution during rheology characterization at different temperatures (calculated via averaged fit) and fitting parameters.

Crosslinking Temperature	η_max_	(Theoretical) Set-on Crosslinking	Slope	Fitting Parameters			
				a	b	c	d
25 °C	(66.83 Pa s) *	23.32 min	0.15 Pa s min^−1^	2.94	−1.11	838.12	0.09
60 °C	369.35 Pa s	1.33 min	0.95 Pa s min^−1^	3.68	−1.11	36.43	0.45
70 °C	246.86 Pa s	−0.67 min	1.28 Pa s min^−1^	3.50	−1.11	3.27	0.63
80 °C	282.34 Pa s	−4.34 min	0.43 Pa s min^−1^	3.56	−1.11	1.07	0.21
90 °C	203.76 Pa s	−4.34 min	0.42 Pa s min^−1^	3.42	−1.11	1.02	0.21

* For 25 °C, maximum value of η at 60 min is displayed; 100% crosslinking was not achieved after this time.

**Table 2:** Values of contraction and mass-specific work of LCEF moving additional weights while being activated at 100 °C in a heating chamber.

	Additional Weights	Contraction	Mass Specific Work
	3.50 g	1.95 ± 0.22%	1.036 ± 0.281 J kg^−1^
	4.71 g	2.72 ± 0.12%	1.919 ± 0.588 J kg^−1^
	5.76 g	2.94 ± 0.22%	2.607 ± 0.803 J kg^−1^
	6.26 g	2.95 ± 0.35%	3.101 ± 1.023 J kg^−1^
	6.76 g	2.83 ± 0.46%	3.279 ± 1.338 J kg^−1^
	7.26 g	2.98 ± 0.76%	3.821 ± 1.793 J kg^−1^
	7.76 g	2.99 ± 0.50%	3.662 ± 1.488 J kg^−1^
	8.26 g	2.43 ± 0.00% *	2.528 ± 0.555 J kg^−1^ *
Max. values	7.14 ± 0.82 g	3.21 ± 0.44%	3.857 ± 1.318 J kg^−1^

* Only a single fiber achieved 8.26 g without breakage; therefore, no standard deviation can be calculated for contraction; standard deviation of specific work was calculated using the Gaussian law of error propagation, including standard deviation of fiber mass and contraction.

## Data Availability

The raw data supporting the conclusions of this article will be made available by the authors on request.
